# Phosphorylation of STAT-1 Serine 727 Is Prolonged in HLA-B27-Expressing Human Monocytic Cells

**DOI:** 10.1371/journal.pone.0050684

**Published:** 2013-01-21

**Authors:** Marja Ruuska, Anna S. Sahlberg, Kaisa Granfors, Markus A. Penttinen

**Affiliations:** 1 Department of Medical Microbiology and Immunology, University of Turku, Turku, Finland; 2 National Institute for Health and Welfare, Department of Infectious Disease Surveillance and Control, Turku, Finland; University of London, St George's, United Kingdom

## Abstract

A tissue antigen, HLA-B27, is strongly associated with a group of rheumatic diseases called spondyloarthritides. Despite the intensive research, the exact role of HLA-B27 in the pathogenesis of these diseases is still unclear. Here we studied whether HLA-B27 modulates the phosphorylation of signal transducer and activator of transcription 1 (STAT-1) serine 727 residue and the localization of STAT-1 in *Salmonella*-infected human monocytic cells. In addition, we studied the role of signaling molecule double-stranded RNA activated protein kinase (PKR) in these modulatory effects. U937 human monocytic cell transfectants stably expressing wild type HLA-B27 or mutated HLA-B27 heavy chains with amino acid substitutions in the B pocket were prepared. The PMA-differentiated cells were infected with *S. enteritidis*. Western blotting was used to detect the phosphorylation of STAT-1, and to visualize the localization of STAT-1 in the cells confocal microscopy was used. Specific inhibitors were employed to study the role of PKR in STAT-1 phosphorylation. We discovered that the phosphorylation of STAT-1 serine 727 is prolonged in cells expressing misfolding forms of HLA-B27 after *S. enteritidis* infection, whereas in mock cells and in cells expressing mutated, non-misfolding HLA-B27 the phosphorylation of serine 727 is transient. Interestingly, STAT-1 serine 727 phosphorylation is partly dependent on PKR. In addition, more STAT-1 is localized in the nucleus of HLA-B27-expressing cells, even before an external trigger, when compared to mock cells. In conclusion, our results show that the phosphorylation of STAT-1 serine 727 residue is prolonged in HLA-B27-expressing monocyte-macrophage U937 cells after bacterial infection. This is of interest since the phosphorylation of serine 727 on STAT-1 is suggested to contribute to macrophage activation and promote inflammatory responses. Therefore, our results provide a mechanism which explains how the expression of an HLA-B27 molecule can impact the course of *Salmonella* infection and reactive arthritis.

## Introduction

An MHC class I tissue antigen, HLA-B27, is strongly associated with a group of rheumatic diseases called spondyloarthritides (SpA), including an acute inflammatory joint disease reactive arthritis (ReA) [1], [2]. Certain gram negative bacteria such as *Salmonella*, *Yersinia*, and *Chlamydia* are known to trigger ReA [3]. There is evidence that triggering bacteria or parts of them can persist for an unusually long time in patients suffering from ReA [4]–[7]. Since most ReA patients are HLA-B27 positive, it is proposed that interaction between host cells and ReA-triggering bacteria might be altered [8].

The mechanism of HLA-B27 for confering disease susceptibility is still unclear. Recent studies suggest that both antigen presenting and non-antigen presenting functions (e.g. dimer formation and the misfolding of HLA-B27 heavy chains [HCs] in the endoplasmic reticulum [ER]) might be involved in the pathogenesis of SpA. [3], [9] Results show that HLA-B27 can misfold in the ER and cause abnormal HC/β2-microglobulin complexes. [10].

The misfolding characteristic seems to be dependent on the amino acid composition of the B pocket, which is a region in the peptide binding groove of HC [11], [12]. The accumulation of misfolded HLA-B27 HCs in the endoplasmic reticulum can lead to ER stress and the activation of the unfolded protein response (UPR) in the cell [10]. Several reports confirm that HLA-B27-expressing cells obtained from transgenic rats exhibit an acute UPR when HLA-B27 is upregulated [13], [14].

We have earlier observed that the elimination of *Salmonella enteritidis* is weakened in U937 monocytic cells transfected with an HLA-B27 molecule, when compared to control cells [15]. More detailed studies indicated that the intracellular replication of *S. enteritidis* is in fact enhanced in HLA-B27-expressing cells, and this phenotype seems to be dependent on HLA-B27 misfolding [16]. In addition, our recent studies showed evidence that p38- and double-stranded RNA activated protein kinase (PKR)-dependent signaling pathways are altered in cells expressing a misfolding HLA-B27 molecule [17], [18].

PKR is capable of forming a complex with a signal transducer and activator of transcription 1 (STAT-1) [19], which is a major mediator of interferon (IFN) signaling [20]. Moreover, it has been reported that PKR is able to control the phosphorylation of STAT-1 [21]. These observations prompted us to study whether PKR-dependent STAT-1 regulation is modulated in B27-expressing U937 cells. We detected earlier that in HLA-B27-expressing cells the phosphorylation of the STAT-1 tyrosine 701 residue is enhanced, even prior to any stimulation, and that this phosphorylation is dependent on PKR activity [22]. In addition to the tyrosine 701 residue– which is necessary for the dimerization of STAT-1, nuclear translocation, and DNA binding– STAT-1 has another important phosphorylation site, serine 727. Mice expressing STAT-1 with a mutation in the serine 727 site are extremely sensitive to bacterial infections and show a strongly reduced expression of IFNγ -induced genes [23]. Moreover, the phosphorylation of STAT-1 serine 727 enhances the full transcriptional activity of STAT-1 [24]. In our previous study we observed that LPS- and *S. enteritidis*-induced phosphorylation of STAT-1 serine 727 tends to be slightly stronger in HLA-B27-expressing cells right after LPS exposure or infection when compared to control cells [22]. The aim of this study was to study the regulation of STAT-1, especially serine 727 phosphorylation, in U937 cells infected with intracellular *S. enteritidis*.

We found that the phosphorylation of STAT-1 serine 727 was prolonged in *Salmonella*-infected U937 human monocytic cells expressing HLA-B27 molecules that have a tendency to misfold during assembly. Studies with a specific PKR inhibitor revealed that the phosphorylation of STAT-1 serine 727 is only modestly dependent on PKR activity in cells expressing misfolding forms of an HLA-B27 molecule, unlike in control cells, at the early time points of infection, but the dependency increased over time. In addition, confocal microscopy studies showed that the localization of STAT-1 in the nucleus was enhanced in HLA-B27-expressing cells. Thus, these alterations in STAT-1 activity might be relevant since it has been reported that the phosphorylation of the STAT-1 serine 727 residue promotes inflammatory responses and is required for the maximal transcriptional activation of STAT-1 [23], [24].

## Materials and Methods

### Cell lines and transfections

The human monocytic cell line U937 was obtained from American Type Culture Collection (ATCC, Rockville, MD). The U937 cell line expresses the HLA class I alleles A3, A26, B18, B51, Cw1, and Cw3 [25]. The cells were cotransfected with HLA-B*2705 genomic DNA (B27g) [26] and pSV2neo vector (to confer resistance to Geneticin [G-418]) by electroporation as described previously [15]. The mutated form of HLA-B*2705 called B27E45M has one amino acid substitution, which means that glutamic acid is changed to methionine at position 45, whereas in the B27H9F mutant, the histidine is changed to phenylalanine at position 9 [12], [27]. For mock transfection, the cells were transfected with pSV2neo (mock) alone. All the transfectants were stable and selected for G-418 resistance and for the surface expression of the transfected molecule, as described previously [16].

The cells were maintained in RPMI 1640 medium supplemented with 10% fetal bovine serum (FBS; PAA Laboratories Pasching, Austria), 1.8 m*M* L-glutamine (Biological Industries, Kibbutz Beit Haemek, Israel), and 50 µg/ml of gentamicin (Biological Industries) at 37°C in a humidified atmosphere of 5% CO_2_. The cell surface expression of the transfected HLA molecules was confirmed by FACScan flow cytometry (BD Immunocytometry Systems, San Jose, CA) each time the new batch of cells was thawed for use. The cells were stained with fluorescein isothiocyanate-conjugated anti-human HLA-B27 monoclonal antibody (mAb) (clone FD705-9EIEI0; One Lambda) as described previously [16]. The level of HLA-B27 expression on the cell surface was found to be comparable in all the HLA-B27-transfected cells as shown earlier [16], and comparable to HLA-B51, one of the MHC class I molecules endogenously expressed by U937 cells.

### PMA stimulation

The cells were diluted to a concentration of 1.0×10^6^/ml and seeded in 25 cm^2^ tissue culture flasks (Greiner Bio One, Frickenhausen, Germany). For cell differentiation to adherent macrophages, the cells were incubated with 10 ng/ml phorbol myristate acetate (PMA; Sigma-Aldrich, St Louis, MO) for 24 hours in RPMI 1640 supplemented with 10% FBS, 1,8 m*M* L-glutamine, and 50 µg/ml gentamicin.

### Bacterial strain

The strain of *S. enteritidis* used was a stool isolate from a patient with *Salmonella*-triggered ReA. Prior to the infection of the cells, the bacteria were grown for 18 hours at 37°C in 10 ml Luria-Bertani broth, then 500 µl of the bacterial culture was transferred into another 10 ml Luria-Bertani broth and incubated for 2 hours to obtain the logarithmic phase of growth [15].

### Infection of cells with *S. enteritidis*

To infect the cells with *S. enteritidis*, the cells were first seeded and then treated with PMA (described above). Two hours before infection, the adherent cells were washed with Hanks' balanced salt solution (HBSS) and the medium was changed to RPMI 1640 supplemented with 10% human AB serum (Finnish Red Cross, Finland). The cells were cocultured with *S. enteritidis* (the multiplicity of infection between 6∶1 and10∶1) for 1 hour at 37°C. The cells were washed 3 times with HBSS, and the incubation medium was changed to supplemented RPMI 1640 (described above) containing 50 µg/ml of gentamicin to kill the remaining extracellular bacteria. The cells were incubated at 37°C until samples were collected at the indicated time points.

### Preparation of cell extracts

After bacterial infection, the samples were harvested and washed twice with ice-cold PBS. The samples were frozen and resuspended in a lysis buffer (420 mM NaCl, 25% glycerol, 1.5 m MgCl_2_, 20 mM HEPES, 0.2 mM EDTA, Complete Mini Protease inhibitor cocktail tablets, and phosSTOP phosphatase inhibitor tablets [1 tablet/ml, Roche Diagnostics, Mannheim, Germany]) for 30 minutes on ice. The samples were centrifuged at 12,000 *g* for 20 minutes at 4°C, and the supernatants were collected as cell extracts containing soluble proteins. The protein concentration was measured by Bradford protein assay (Bio-Rad, Hercules, CA).

### Gel electrophoresis and Western blot analysis

The cell extracts (35 µg of protein) in a Laemmli buffer were subjected to 7% sodium dodecyl sulphate polyacrylamide gel electrophoresis (SDS-PAGE) and transferred to nitrocellulose filters (Protran Nitrocellulose; Schleicher & Schuell, Keene, NH) using a semidry transfer apparatus (Bio-Rad). The Western blot analysis was performed using rabbit polyclonal antibodies (pAb) STAT-1 (1∶1000; 9172; Cell Signaling Technology, Danvers, MA) and phospho-STAT-1 (Ser727) (1∶800; 9177; Cell Signaling Technology). Rat monoclonal antibody (mAb) Hsc70 (1∶10 000; SPA-815; Stressgen Bioreagents, Farmingdale, NY) was used as a loading control. Horseradish peroxidase-conjugated anti-rabbit antibody and anti-rat antibody were purchased from Promega (Fitchburg, WI) and Stressgen Bioreagents, respectively. The blots were developed using an enhanced chemiluminescence method (Millipore, Billerica, MA). To quantify the intensity of the bands Image J image processing software was used. The relative intensity of the bands was calculated as the intensity of the phosphorylated band divided by the intensity of the respective band of a loading control (Hsc70) and normalized to the control “C” (given value one). The statistical analysis was performed using Student's two-tailed t-test.

### Inhibition assays

The PKR inhibitor PKR+, (10 µ*M*, Calbiochem, Billerica, MA) and a negative control for the PKR inhibitor PKR−, (10 µM, Calbiochem) were added to the tissue culture flasks after external bacteria were washed away. The assay was continued as in the *S. enteritidis* infection (described above).

### Confocal microscopy

13-mm round glass cover slips were placed onto the 24-well tissue culture plates. The cells were diluted to a concentration of 1–2×10^6^/ml and seeded into the 24-well plates. The cells were first PMA-maturated, infected with *S. enteritidis* and subjected to PKR inhibition as described above. At 2 hours post infection, the cells were fixed with 3.7% formaldehyde, permeabilized with 100% ice cold methanol and stained with STAT-1 antibody (1∶50; Cell Signaling Technology) and Alexa fluor 568 secondary antibody (1∶800; Invitrogen life technologies, Carlsbad, CA). The nuclei were stained with Hoechst. The cells were visualized by a Zeiss LSM510 META laser scanning microscope with a 100× oil objective (Carl Zeiss, Jena, Germany). The statistical analysis of the confocal images was performed using an Image J image processing program. The amount of STAT-1 in the nucleus was analyzed by measuring the amount of red colour (STAT-1) in the defined area of the blue stained nuclei. The relative amounts of nuclear STAT-1 are weighted averages of three independent experiments.

## Results

To study the effect of the HLA-B27 expression on STAT-1 serine 727 phosphorylation we used human monocytic U937 cells transfected with genomic DNA encoding HLA-B27 (B27g). For control, the cells were transfected with pSV2neo vector (mock) alone. To investigate whether the tendency of HLA-B27 HCs to misfold affects STAT-1 phosphorylation, we used cells expressing mutated HLA-B27 molecules. In the B27E45M mutant, the methionine at position 45 in the B pocket, which is a region of the peptide-binding groove, is substituted for glutamic acid. It has been shown that the glutamic acid at this position is crucial for the aberrant behaviour of the B27 molecule, and that the substitution with methionine radically enhances folding and prevents the misfolding of the HCs. [12], [16]. The B27H9F mutant, which misfolds even more easily than the wild type HLA-B27 [16], was used to further confirm the role of misfolding. In the B27H9F mutant, the histidine is changed to phenylalanine at position 9 in the B pocket of HLA-B27 [27].

### Phosphorylation of STAT-1 serine 727 is prolonged in cells expressing misfolding forms of HLA-B27

**Figure 1:**
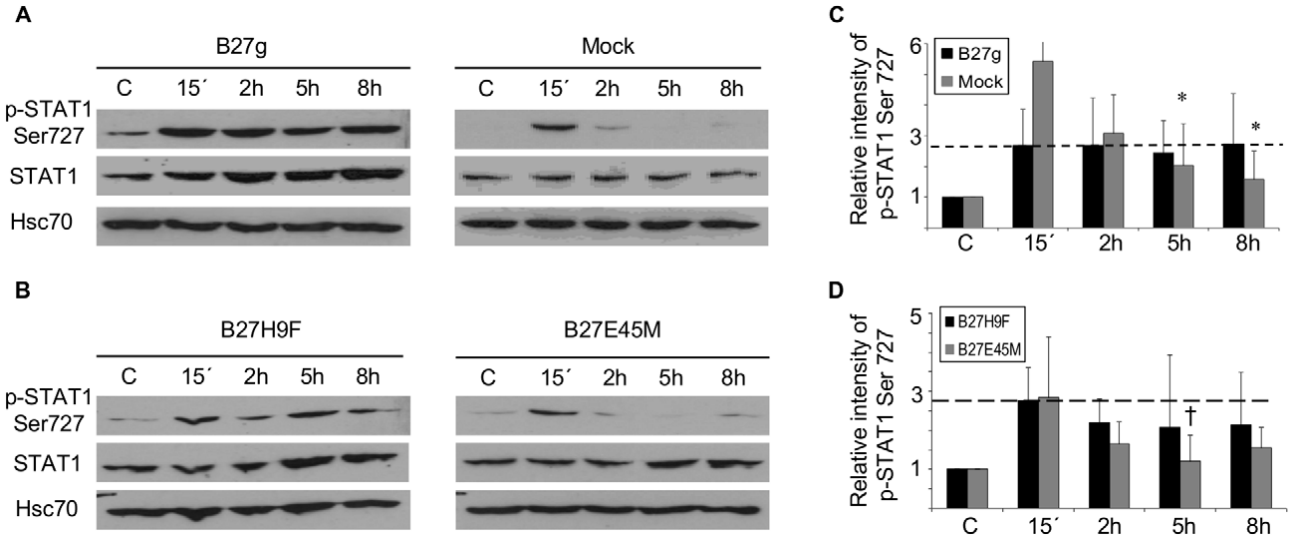
Prolonged phosphorylation of STAT-1 serine 727 in cells expressing misfolding forms of HLA-B27 molecule. U937 transfectants were PMA-maturated, infected with *Salmonella enteritidis*, and harvested at the indicated time points after excess bacteria were washed away. The phosphorylation of STAT-1 serine 727 was studied by Western blot method with p-STAT-1 Ser727 antibody. Cell extracts (35 µg of each) were loaded on SDS-page gel. **A**, The representative figure of U937 cells transfected with genomic clones of HLA-B27 (B27g) or with vector alone (mock). “C” denotes uninfected cells. **B**, The representative figure of U937 cells transfected with mutated, misfolding form of HLA-B27 (B27H9F) or with mutated, non-misfolding form of HLA-B27 (B27E45M). **C**, The relative intensity of STAT-1 serine 727 phosphorylation in B27g-expressing cells and in mock cells. The bars show the mean ± SD of 6 independent experiments. **D**, The relative intensity of STAT-1 serine727 phosphorylation in B27H9F- and B27E45M-transfected cells. The bars show the mean ± SD of 5 independent experiments. Blots were stripped and reprobed with STAT-1 antibody and Hsc70 antibody as a loading control. The relative intensity of p-STAT1 Ser 727 was calculated as the intensity of the phosphorylated band divided by the intensity of the respective loading control and normalized to the uninfected control (C, which is given value one). * = *P*<0.05 and † = *P*<0.07 versus 15 min time point of the respective cell line.

We have earlier observed that in HLA-B27-expressing U937 monocytic cells the phosphorylation of STAT-1 serine 727 is slightly enhanced shortly after LPS stimulation or *S. enteritidis* infection when compared to mock or B27E45M-transfected cells [22]. This prompted us to study the phosphorylation of STAT-1 serine 727 and particularly the role of the misfolding of HLA-B27 HCs in STAT-1 phosphorylation, in *S. enteritidis*-infected cells. The intracellular infection of *S.enteritidis* in U937 cells transfected with HLA-B27 was studied earlier, which included measuring a number of living intracellular bacteria and reporting them as a colony forming unit (CFU); microscopy was used to further demonstrate the intracellular infection inside the cells [15], [16]. As seen in Figure 1 (A and B), a modestly increased phosphorylation of STAT-1 serine 727 was observed in all the HLA-B27-transfected cells before infection (time point C), when compared to the mock cells. According to quantification, the phosphorylation level of STAT-1 serine 727 was two times higher in the B27g transfectant and approximately 1.7 times higher in the B27E45M and B27H9F transfectants, when compared to mock (data not shown). Besides mock, the HLA class I molecule, HLA-A2, which is not associated with ReA, was used as a control. The phosphorylation levels of STAT-1 serine 727 in the HLA-A2-transfected cells and in the mock cells were close to equal before infection (data not shown). *Salmonella* infection induced STAT-1 serine 727 phosphorylation in all the HLA-B27-transfected cells as well as in the mock cells. However, in cells expressing misfolding forms of HLA-B27 (the B27g and B27H9F transfectants), the phosphorylation of STAT-1 serine 727 persisted high even at later time points (5 h and 8 h) and close to the level observed at 15 minutes time point, while in the mock and B27E45M-transfected cells the phosphorylation of STAT-1 serine 727 was transient and decreased near the level observed before infection (time point C) in time (Figure 1). This indicates that the activation of STAT-1 serine 727 is prolonged in cells expressing misfolding HLA-B27 HCs. This is of interest since STAT-1 serine 727 phosphorylation is reported to be required for the maximal transcriptional activity of STAT-1 and also to promote inflammatory responses [23], [24]

### STAT-1 serine 727 phosphorylation is partially dependent on PKR activity

**Figure 2:**
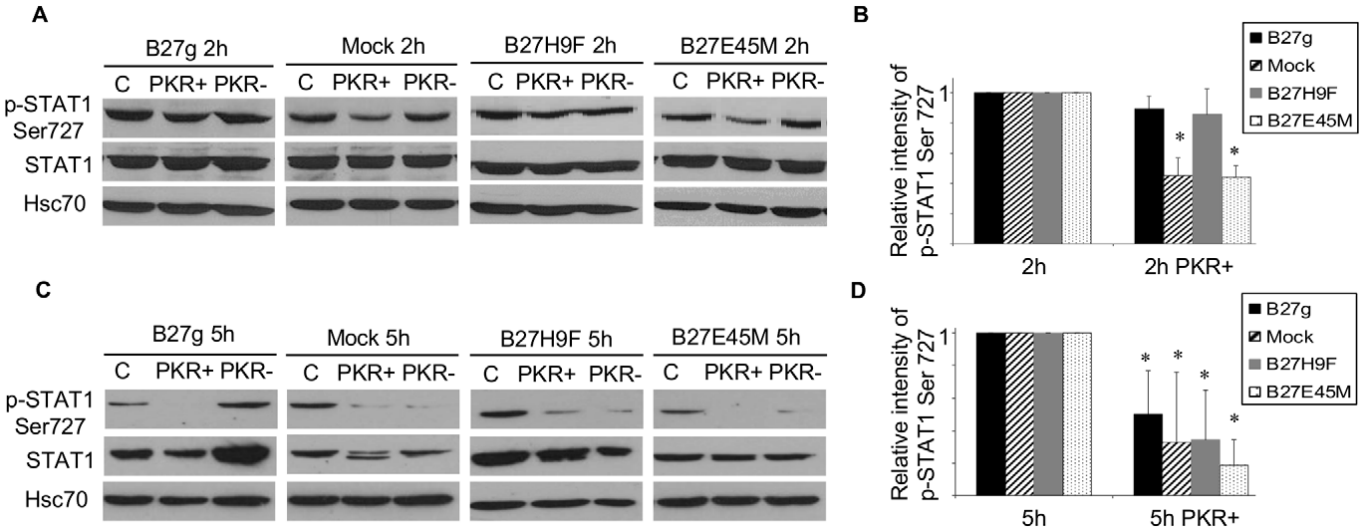
Phosphorylation of STAT-1 serine 727 is only partially dependent on PKR activity. U937 transfectants were PMA-maturated, infected with *S. enteritidis*, and treated with a PKR inhibitor (PKR+) or an inhibitor control (PKR−). Cells were harvested 2 hours (A and B) or 5 hours (C and D) after excess bacteria were washed away. The Phosphorylation of STAT-1 serine 727 was studied by Western blot method with p-STAT-1 Ser727 antibody. Cell extracts (35 µg of each) were loaded on SDS-page gel. **A**, The representative figure at 2 hour time point of U937 cells transfected with genomic clone B27g, vector (mock), misfolding B27H9F or with non-misfolding B27E45 mutants. “C” denotes control *S. enteritidis*-infected cells without PKR-treatment. **B**, The relative intensity of STAT-1 serine 727 phosphorylation after PKR inhibition at 2 hour time point. The bars show the mean ± SD of 3 independent experiments. **C**, The representative figure at 5 hour time point of U937 cells transfected with genomic clone B27g, vector (mock), B27H9F or with B27E45 mutants. **D**, The relative intensity of STAT-1 serine 727 phosphorylation after PKR inhibition at 5 hour time point. The bars show the mean ± SD of 4 independent experiments. The blots were stripped and reprobed with STAT-1 antibody and Hsc70 antibody as a loading control. The relative Intensity values were normalized to the *S. enteritidis*-infected control (C, which is given value one). * = *P*<0.05 versus 2 h/5 h of the respective cell line.

Since it has been reported that PKR is able to regulate the phosphorylation of STAT-1 [22], [28], we wanted to see whether the inhibition of PKR affects the *Salmonella*-induced phosphorylation of STAT-1 serine 727 residue in our HLA-B27-transfected U937 cells. We used a specific PKR inhibitor (PKR+) that blocks the ATP-binding site on PKR thereby preventing the autophosphorylation of PKR. We have shown earlier that the PKR inhibitor effectively blocks the PKR phosphorylation in our U937 cells that are transfected with HLA-B27 [18]. As seen in Figures 2A and 2B, in the mock and non-misfolding B27E45M-transfected cells the phosphorylation of STAT-1 serine 727 is significantly decreased as a result of the PKR inhibition 2 hours after infection. Interestingly, in the B27g-expressing cells and misfolding B27H9F-transfected cells, the inhibition of PKR has only a modest effect on STAT-1 serine727 phosphorylation (Figures 2A and 2B). This is in line with our earlier observation: the same phenomenon was detected in the LPS-stimulated mock and B27g-transfected U937 monocytic cells [22]. However, at a later time point (5 h after infection), the inhibition of PKR caused a significant decrease in STAT-1 serine 727 phosphorylation in all the cell lines, including the B27g- and B27H9F-transfected cells (Figures 2C and 2D).

### More STAT-1 is localized in the nucleus of HLA-B27-expressing cells

**Figure 3:**
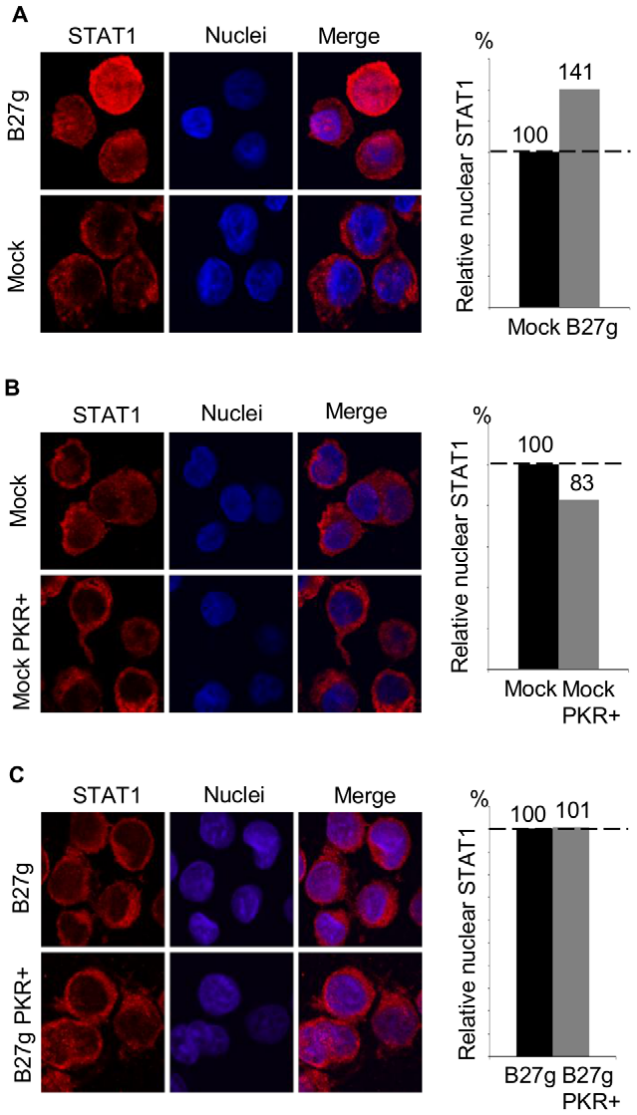
Nuclear localization of STAT-1 and the influence of PKR on STAT-1 localization. **A**, The confocal microscopy images of the STAT-1 localization in uninfected U937 B27g-expressing cells compared to mock cells. The relative amounts of nuclear STAT-1 of uninfected B27g cells vs. uninfected mock cells (n = 136 cells of each) are represented in the diagram. **B**, The confocal microscopy images of the STAT-1 localization of *S. enteritidis*-infected U937 mock-transfected cells. In the lower panel, the cells were treated with the PKR inhibitor and all the cells were collected 2 hours after excess bacteria were washed away. The relative amounts of nuclear STAT-1 of *S. enteritidis-*infected mock vs. mock PKR+ (n = 88 cells of each) are shown in the diagram. **C**, The confocal microscopy images of the STAT-1 localization of *S. enteritidis*-infected U937 B27g-transfected cells. In the lower panel, the cells were treated with the PKR inhibitor and all the cells were collected 2 hours after excess bacteria were washed away. The diagram displays the relative amounts of nuclear STAT-1 of *S. enteritidis*-infected B27g vs. B27g PKR+ (n = 73 cells of each). The amount of nuclear STAT-1 was calculated by measuring the amount of red colour (STAT-1) in the defined area of the blue stained nuclei. The relative amounts are weighted averages of 3 independent experiments. (PKR+ = cells treated with the PKR inhibitor.)

As a transcription factor, STAT-1 has to be localized in the nucleus where it can bind to DNA [29]. Since we have earlier detected that STAT-1 tyrosine 701 phosphorylation, which is needed for nuclear localization, is enhanced in cells expressing misfolding forms of HLA-B27 [22], and because we now observed that the phosphorylation of serine 727 residue is prolonged in these cells ensuring the maximal transcriptional activity, we used confocal microscopy to study the localization of STAT-1 in HLA-B27-positive cells. As shown in Figure 3A, more STAT-1 can be detected in the nucleus of B27g-transfected cells, even before any external triggers (LPS or *S. enteritidis*), when compared to the mock cells. This suggests that the enhanced phosphorylation of STAT-1 observed in HLA-B27-expressing cells is functionally relevant (Figure 3A). Moreover, the quantification studies showed that there is approximately 40% more STAT-1 in the nucleus of B27g-transfected cells than in that of the mock cells (Figure 3A).

Since we have detected differences in the PKR dependency on STAT-1 phosphorylation between the cell lines at 2 hour time point, we were interested to see whether the inhibition of PKR also affects the localization of STAT-1. Further studies with microscopy revealed that the inhibition of PKR effectively restrains the STAT-1 nuclear localization in mock cells (Figure 3B) because, according to quantification, there is ∼17% less STAT-1 in the nucleus of the cells in which the PKR inhibitor was used (Figure 3B). In contrast, in HLA-B27-expressing cells the inhibition of PKR did not prevent the STAT-1 nuclear localization (Figure 3C).

## Discussion

In this study, we aimed to examine whether the phosphorylation of serine 727 residue of STAT-1 is altered in U937 cells expressing HLA-B27 upon *Salmonella enteritidis* infection and furthermore, whether the phosphorylation is dependent on PKR activity. We also studied the localization of STAT-1 in U937 cells. In brief, we observed that the phosphorylation of STAT-1 serine 727 is prolonged in cells expressing misfolding forms of an HLA-B27 molecule after *S. enteritidis* infection. Studies using a specific PKR inhibitor suggest that the *S. enteritidis*-induced STAT-1 serine 727 phosphorylation is partly dependent on PKR signaling. Localization studies using microscopy revealed that more STAT-1 is found in the nucleus of HLA-B27-expressing cells when compared to mock cells.

STAT-1 is a critical mediator of IFN-induced gene responses, and its function is regulated through the phosphorylation of two distinct phosphorylation sites, tyrosine 701 and serine 727 [20]. Because STAT-1 is an important mediator of interferon signaling [20], we wanted to study the secretion of IFN-γ, IFN-β and also IL-12. However, our U937 monocytic cells did not secrete a measurable amount of these cytokines (data not shown). Nevertheless, this does not rule out the possibility that a prolonged activation of STAT-1 may alter the expression of other STAT-1 dependent genes in HLA-B27-expressing cells. Further studies are required to elucidate this question. In addition to type I or type II interferon triggered stimulation, other factors, such as UV treatment or lipopolysacharide, can activate serine 727 phosphorylation [20]. It has been reported that the stress-activated phosphorylation of serine 727 residue is mediated through the p38 mitogen-activated protein kinases pathway [30]. Our observations, however, suggest that in U937 cells, the activation of STAT-1 (the activation of serine 727 and tyrosine 701) is mainly PKR-dependent [22]. This is of interest, because our previous study shows that PKR is overexpressed and hypophosphorylated in cells expressing misfolding HLA-B27 HCs [18]. Therefore, our results suggest that the altered regulation of STAT-1 observed in HLA-B27-expressing cells is dependent on the modulation of PKR function induced by misfolding HLA-B27 HCs.

The accumulation of misfolded HLA-B27 HCs in the ER can lead to ER stress and the activation of the UPR in the cell [10]. However, we have not been able to detect signs of UPR activation in stably-transfected cells expressing HLA-B27, including the U937 cells studied here [16]. In contrast, we have observed an altered expression and the activation of PKR in HLA-B27-expressing U937 cells [18]. There is evidence that PKR can function as an important sensor for cells to adapt for chronic ER stress [31]. Therefore, the modulation of the PKR function may be obligatory for U937 cells that have been selected for survival while expressing misfolding proteins to manage chronic ER stress. In addition, the altered PKR-dependent STAT-1 activation may function as a compensatory mechanism to protect the cells from dying. Recent evidence suggests that macrophages from AS patients have a modulatory inflammatory response upon LPS stimulation although no signs of UPR were detected [32]. Further studies are required to study whether PKR is modulated in macrophages obtained from HLA-B27 patients suffering from SpA.

The Th17/IL-23 axis plays an important role in the development of chronic inflammation and in the host defence against bacterial infections [33]. For example, in AS patients the number of IL-17 secreting Th17 cells is greater and the level of IL-17, which is associated with inflammation and autoimmunity, is higher [34]. Also, patients with AS have a higher serum level of IL-23, which is essential for the differentiation of Th17 lymphocytes [35]. In addition, HLA-B27 misfolding and UPR have been shown to enhance IL-23 production in transgenic rats [36]. IL-23 binding to an IL-23 receptor triggers the activation of Janus family kinase (Jak2) and tyrosine kinase 2 (Tyk2), which in turn phosphorylates STAT family members, including STAT-1 [33]. Thus, it is possible that the enhanced and prolonged STAT-1 phosphorylation in HLA-B27-positive cells might be in part the consequence of enhanced IL-23 production. Further studies are required to elucidate this question.

We have earlier reported that in LPS-stimulated HLA-B27-expressing cells the inhibition of PKR blocks tyrosine 701 phosphorylation effectively, but the phosphorylation of serine 727 stayed relatively high after the PKR inhibition when compared to mock cells [22]. Hence, we wanted to study how the inhibition of PKR affects STAT-1 serine 727 phosphorylation after *Salmonella enteritidis* infection. Interestingly, results indicated that 2 hours after infection, the phosphorylation of serine 727 was only modestly inhibited by the PKR inhibitor in the HLA-B27-expressing cells, whereas in the mock cells, the inhibition was more effective (Figure 2A and 2B). In contrast, at 5 hour time point phosphorylation was effectively inhibited in all the cell lines studied (Figure 2C and 2D). Although one explanation could be that shortly after infection the phosphorylation of serine is mediated through the p38 signaling pathway, we have not detected changes in serine 727 phosphorylation after the p38 inhibition (data not shown). A more plausible explanation is that the altered PKR regulation in HLA-B27 cells modulates the interaction between STAT-1 and PKR leading to a prolonged activation of serine 727 upon *Salmonella* infection. The phosphorylation of Serine 727 is important for the maximal transcriptional activity of STAT-1 and therefore, the prolonged activity of this phosphorylation site may induce an exaggerated inflammatory response in *Salmonella*-infected HLA-B27 cells. In fact, HLA-B27-positive individuals suffering from *Salmonella* infection have more severe symptoms [37]. On the other hand, STAT-1 also plays an important role in bacterial clearance because mice expressing STAT-1 with a mutated 727 residue were shown to be highly sensitive to bacterial infections [23]. Furthermore, a STAT-1 defect increases the risk of invasive salmonellosis in humans [38].

The latent inactivated STAT-1 resides in the cytoplasm, but as a transcription factor STAT-1 exerts its biological function in the nucleus. STAT-1 is localized to the nucleus after it is activated by tyrosine 701 phosphorylation and dimerization [29], and we have earlier shown that in HLA-B27-expressing cells STAT-1 tyrosine phosphorylation is enhanced [22]. Now, our localization studies with confocal microscopy revealed that in HLA-B27-expressing cells more STAT-1 is localized in the nucleus than in mock cells even before *S. enteritidis* infection or LPS stimulation (Figure 3A). This finding supports our previous assumption that the expression of the misfolding HLA-B27 molecule alone, without any external trigger, is sufficient to cause the functional activation of STAT-1 in U937 cells. Furthermore, we observed that in HLA-B27-expressing cells the PKR inhibitor did not significantly inhibit STAT-1 serine 727 phosphorylation 2 hours after infection, whereas a clear inhibition was observed in mock cells (Figure 2). Interestingly, studies with microscopy showed that the PKR inhibitor did not prevent the nuclear localization at the same time point in HLA-B27 cells. In contrast, a clear inhibition was observed in mock cells. These results together suggest that STAT-1 serine 727 may have an important function in the intracellular localization of STAT-1 in U937 cells.

Monocytes/macrophages play a major role in inflammation and immune responses, whereas STAT-1 is a major activator in macrophage activation [39]. Interferons or stress signals are known to induce the phosphorylation of the STAT-1 serine 727 residue, which is needed for the maximal activation of STAT-1 [24]. Here, in this study we detected a prolonged phosphorylation of STAT-1 serine727 in cells expressing HLA-B27. Since studies suggest that the STAT-1 serine 727 phosphorylation promotes inflammatory responses [23], our finding provides a mechanism explaining how intracellular *Salmonella* infection may induce an exaggerated inflammatory response in HLA-B27-expressing cells.
